# Discoid lupus erythematosus of the palms: A case report

**DOI:** 10.1002/ccr3.5048

**Published:** 2022-04-22

**Authors:** Albert E. Zhou, Gibran Shaikh, Marcia S. Driscoll

**Affiliations:** ^1^ Department of Dermatology University of Maryland School of Medicine Baltimore Maryland USA

**Keywords:** autoimmune, discoid, lupus, palms, plaques

## Abstract

Discoid lupus erythematosus is an autoimmune connective‐tissue disease that represents a subset of conditions on the cutaneous lupus spectrum. The lesions are characterized by disk‐shaped plaques on photo‐exposed skin with inflammatory hyperpigmentation and adherent scale. Here, we present a patient with a rare manifestation of discoid lesions on the palms.

## Graphical abstract

Discoid lupus erythematosus is an autoimmune connective‐tissue disease that is typically characterized by disk‐shaped plaques on photo‐exposed skin. Discoid lesions on the bilateral palms are rare and may be refractory to conventional treatments.

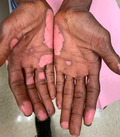

## 1 CASE

**FIGURE 1:**
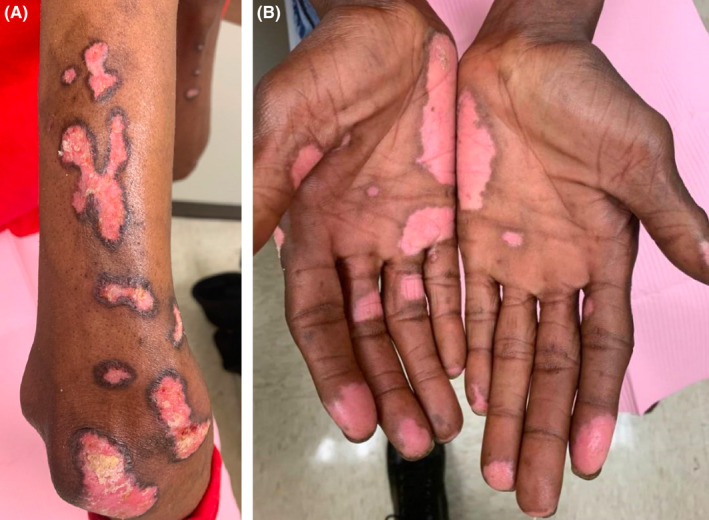
Scaly erythematous to violaceous thin plaques, some with hyperpigmented rims most prominently on the extensor arms (A) and palmar surface of hands (B)

A 50‐year‐old man with hepatitis C presented to the clinic complaining of a progressive, mildly pruritic eruption on his ears, face, and bilateral upper extremities over the past few years (Figure 1A). He believes these lesions are associated with his occupation as a janitor and repeated exposures to various chemical solutions. Examination revealed scaly erythematous to violaceous thin plaques, some with hyperpigmented rims most prominently on the extensor arms, nasal ala, bilateral concha bowls, and palms (Figure 1B). A 5‐mm punch biopsy demonstrated epidermal and follicular interface dermatitis along with basement zone thickening and mucin deposition. Anti‐nuclear antibodies were positive (1:320), and direct immunofluorescence revealed thick granular deposition of IgM and C3 along the epidermal basement membrane. A formal diagnosis of discoid lupus erythematosus was made. Our patient was advised on regular, high SPF sunscreen use and given high‐potency topical steroids but was lost to follow‐up before the initiation of systemic treatment or further workup for systemic disease.

## 2 DISCUSSION

Discoid lupus erythematosus (DLE) is an autoimmune disease typically characterized by lesions on photo‐exposed skin. It represents a subset of conditions on the cutaneous lupus spectrum, and up to 15% of patients will meet criteria for systemic lupus erythematosus.^1^ DLE can occur in all age groups, often between the second and fourth decades of life, and more frequently in women and African Americans.^1^ The incidence in Europe and the United States is estimated to be 4 per 100,000 people.^2^

The pathogenesis of this connective‐tissue disease is multifactorial and has not been fully elucidated. In genetically susceptible individuals, exposure to ultraviolet (UV) radiation, medications, or cigarette smoke can cause a loss of immune tolerance, DNA damage, and dysregulated apoptosis.^1^ This triggers an inflammatory cascade of cytokine activation, notably the type I and type II interferons, leading to the formation and deposition of autoantibodies and immune complexes.^3^, ^4^

The disk‐shaped plaques are well‐demarcated with inflammatory hyperpigmentation and adherent scale that can extend into dilated hair follicles (follicular plugging).^5^ Removal of the scale can reveal keratotic spikes (“carpet tack sign”).^1^ Lesions are most often located on the face, ears, and scalp. Extensive disease beyond the head and neck is predicative of systemic disease.^1^ In our case, discoid lesions on the bilateral palmar surfaces represent a distinct rarity that occurs in less than 2% of patients.^3^, ^6^, ^7^ The absence of hair follicles on the volar skin does not preclude the development of classic features of DLE.^7^ Other reports have suggested that lesions of the palms and soles may be refractory to conventional forms of treatment.^8^, ^9^ Future research could elucidate whether there are differences in treatment response between lesions of the palms and those of the sun‐exposed sites.

Without treatment, scarring, atrophy, and hypopigmentation can cause permanent disfigurement.^5^ Scarring alopecia and squamous cell carcinoma can develop in longstanding discoid.^1^ Providers are encouraged to perform a thorough review of systems to evaluate for systemic lupus erythematous and assess skin lesions with the Cutaneous Lupus Erythematosus Disease Area and Severity Index (CLASI), a tool developed to score the disease activity (the presence of erythema, scale, and hypertrophy) and damage (dyspigmentation, scarring, and atrophy).^5^, ^10^ Despite being primarily a clinical diagnosis, biopsy can be helpful in unclear cases. Pathology will reveal vacuolar degeneration of the basal cell layer, mononuclear cell infiltrate, necrotic keratinocytes, and thickening of the basement membrane with destruction of the sebaceous glands and elastic fibers throughout the reticular dermis.^1^ Direct immunofluorescence will show a continuous band of immunoreactants deposited along the dermal‐epidermal junction, commonly known as the “lupus band test.”^11^ Tissue cultures are usually unrevealing.

Treatment of DLE centers on high‐potency topical steroids, topical calcineurin inhibitors, and systemic antimalarial therapy (*ie*, hydroxychloroquine) with the goal of minimizing scarring.^5^ Methotrexate, mycophenolate mofetil, and acitretin are options for refractory disease, although newer systemic immunosuppressive medications are being introduced.^5^ Patient education on photoprotection, including the liberal use of zinc oxide chemical sunscreens, is a fundamental aspect in treatment, especially in patients with skin of color who may not be accustomed to wearing sunscreen. Smoking cessation is another cornerstone in minimizing disease activity and improving efficacy of antimalarial therapeutics. Vitamin D supplementation is also recommended.^12^ Patients may benefit from a referral to a rheumatologist for interdisciplinary management and further workup for systemic disease.

Differential diagnoses of DLE include lupus pernio and neurotic excoriations. Lupus pernio is a cutaneous manifestation of sarcoidosis, having a similar clinical distribution as DLE, but characterized histologically by the presence of noncaseating granulomas. These patients should undergo a chest X‐ray to evaluate for any pulmonary manifestations. Patients with neurotic excoriation may require a referral to psychiatry for behavioral modification therapy and initiation of a serotonin‐specific reuptake inhibitor.

The patient in this case presented with discoid lupus erythematosus with lesions on the bilateral palms, a distinct and rare phenomenon, which may be refractory to conventional treatment options. Large, controlled clinical trials should investigate treatment differences between lesion locations and establish the efficacy among various therapies for these patients.

## CONFLICTS OF INTEREST

The authors have no conflicts of interest to declare.

## AUTHOR CONTRIBUTIONS

AEZ drafted, wrote, and revised the manuscript. GS conceived the idea and revised the manuscript. MSD was responsible for the disgnosis of the condition and subsequent patient management, and offered suggestions and feedback for the manuscript.

## ETHICAL APPROVAL

*Studies Involving Human Subjects*: Ethics approval is not required for anonymized case reports.

## CONSENT

We received written informed consent from the participant for the publication of their medical case and any accompanying images.

## Data Availability

All data generated or analyzed during this study are included in this article. Further enquiries can be directed to the corresponding author.

## References

[1] McDaniel B , Sukumaran S , Koritala T , et al. Discoid Lupus Erythematosus. [Updated September 4, 2021]. In: StatPearls [Internet]. Treasure Island (FL): StatPearls Publishing; 2021. https://www.ncbi.nlm.nih.gov/books/NBK493145/ 29630197

[2] Durosaro O , Davis MDP , Reed KB , Rohlinger AL . Incidence of cutaneous lupus erythematosus, 1965–2005: a population‐based study. Arch Dermatol. 2009;145(3):249‐253. 10.1001/archdermatol.2009.21 19289752 PMC3953616

[3] Uva L , Miguel D , Pinheiro C , Freitas JP , Marques Gomes M , Filipe P . Cutaneous manifestations of systemic lupus erythematosus. Autoimmune Dis. 2012;2012:834291. 10.1155/2012/834291 22888407 PMC3410306

[4] Achtman JC , Werth VP . Pathophysiology of cutaneous lupus erythematosus. Arthritis Res Ther. 2015;17(1):182. 10.1186/s13075-015-0706-2 26257198 PMC4530484

[5] Garza‐Mayers AC , McClurkin M , Smith GP . Review of treatment for discoid lupus erythematosus. Dermatol Ther. 2016;29(4):274‐283. 10.1111/dth.12358 27073142

[6] Rebora A , Bardelli A , Parodi A . Discoid lupus erythematosus of the palms. J Dermatol. 1985;12(2):195‐197. 10.1111/j.1346-8138.1985.tb01559.x 3897328

[7] Parish LC , Kennedy RJ , Hurley HJ . Palmar lesions in lupus erythematosus. Arch Dermatol. 1967;96(3):273‐276. 10.1001/archderm.1967.01610030051008 6038753

[8] Goyal S , Nousari HC . Treatment of resistant discoid lupus erythematosus of the palms and soles with mycophenolate mofetil. J Am Acad Dermatol. 2001;45(1):142‐144. 10.1067/mjd.2001.114297 11423853

[9] Ashinoff R , Werth VP , Franks AG . Resistant discoid lupus erythematosus of palms and soles: successful treatment with azathioprine. J Am Acad Dermatol. 1988;19(5):961‐965. 10.1016/S0190-9622(88)70259-5 3192780

[10] Klein R , Moghadam‐Kia S , LoMonico J , et al. Development of the CLASI as a tool to measure disease severity and responsiveness to therapy in cutaneous lupus erythematosus. Arch Dermatol. 2011;147(2):203. 10.1001/archdermatol.2010.435 21339447 PMC3282059

[11] Reich A , Marcinow K , Bialynicki‐Birula R . The lupus band test in systemic lupus erythematosus patients. Ther Clin Risk Manag. 2011;7:27‐32. 10.2147/TCRM.S10145 21339940 PMC3039011

[12] Hassanalilou T , Khalili L , Ghavamzadeh S , Shokri A , Payahoo L , Bishak YK . Role of vitamin D deficiency in systemic lupus erythematosus incidence and aggravation. Auto Immun Highlights. 2017;9(1):1. 10.1007/s13317-017-0101-x 29280010 PMC5743852

